# Assessment of precision and reproducibility of a new myograph

**DOI:** 10.1186/1475-925X-6-49

**Published:** 2007-12-20

**Authors:** Niels Rahe-Meyer, Michael Winterhalter, Aminul I Ahmed, Christian Weilbach, Matthias Gross, Siegfried Piepenbrock, Matthias Pawlak

**Affiliations:** 1 Department of Anesthesiology, Hannover Medical School, Carl-Neuberg-Str. 1, D-30625 Hannover, Germany; 2 Department of Neurosurgery, Wessex Neurological Centre, Southampton University Hospitals NHS Trust, Southampton S016 6YD, UK; 3 Department of Anesthesiology, St. Josefs Stift Cloppenburg, Krankenhausstr. 13, D-49661 Cloppenburg, Germany; 4 Department of Physiology, Biochemistry and Hygiene, University School of Physical Education, ul. Królowej Jadwigi 27/39, Poznań, Poland; 5 Institute of Physiology, University of Wuerzburg, Roentgenring 9, D-97070 Wuerzburg, Germany

## Abstract

**Background:**

The physiological characteristics of muscle activity and the assessment of muscle strength represent important diagnostic information. There are many devices that measure muscle force in humans, but some require voluntary contractions, which are difficult to assess in weak or unconscious patients who are unable to complete a full range of voluntary force assessment tasks. Other devices, which obtain standard muscle contractions by electric stimulations, do not have the technology required to induce and measure reproducible valid contractions at the optimum muscle length.

**Methods:**

In our study we used a newly developed diagnostic device which measures accurately the reproducibility and time-changed-variability of the muscle force in an individual muscle. A total of 500 *in-vivo *measurements of supra-maximal isometric single twitch contractions were carried out on the *musculus adductor pollicis *of 5 test subjects over 10 sessions, with ten repetitions per session. The same protocol was performed on 405 test subjects with two repetitions each to determine a reference-interval on healthy subjects.

**Results:**

Using our test setting, we found a high reproducibility of the muscle contractions of each test subject. The precision of the measurements performed with our device was 98.74%. Only two consecutive measurements are needed in order to assess a real, representative individual value of muscle force. The mean value of the force of contraction was 9.51 N and the 95% reference interval was 4.77–14.25 N.

**Conclusion:**

The new myograph is a highly reliable measuring device with which the *adductor pollicis *can be investigated at the optimum length. It has the potential to become a reliable and valid tool for diagnostic in the clinical setting and for monitoring neuromuscular diseases.

## Background

In both men and animals, the correct wiring of the nervous system to discrete motor units during development determines the functional outcome of the muscle as assessed by the contraction force. Some traditional methods of patient evaluation, including neurological assessment, manual muscle testing and rating scales, involve subjective elements and lack the sensitivity and reproducibility needed to detect small but meaningful changes in the progression of the disease, reflecting deterioration or therapeutic efficacy. In diseases affecting the neuromuscular unit, the objective assessment of the quality of the muscle's contraction force for individual patients is made using a variety of electrophysiological tests including electromyography (EMG) and nerve conduction recordings. The assessment of these physiological characteristics generates a clear topographical representation of the spread of the disease and enables the monitoring of the progression of nerve-muscle diseases. However, in the clinical setting, the EMG devices have limitations in the diagnosis of myopathies, since they require a conscious and compliant patient and the recorded potentials do not directly correlate with muscle strength.

Manual testing without any measuring device is the standard method of assessment of muscle strength in most clinics. This method has certain disadvantages [1] because it relies on subjective assessment by the clinician and is thus inadequate to quantify small changes in muscle strength.

Several objective methods for the study of muscle force in humans, using a diverse range of devices to measure the contraction of the muscle, have been developed and are applied in medicine and medical science. However, these devices have their own limitations that make them a less than ideal clinical diagnostic device. Some measure only maximal voluntary contractions [1-9], which is sub-optimal since patients are often too weak and unable to complete a full range of motivation in voluntary force assessment tasks. Moreover, these devices rely on voluntary contractions, cannot examine individual muscles and have no pre-stretching mechanism to bring the muscles to their optimal length. Therefore the position of the extremity and the length of the muscle depend on the characteristics of the measuring equipment. Although good reproducibility may be achieved [3,6,7,9], the results cannot lead to absolute, valid statements on the examined muscle, independent of measurement conditions (which include variable patient compliance, undefined summation of muscle vectors and artificial pre-stretching position).

Devices which rely upon electrically stimulated standard contractions are characterized by inadequately defined axle-adjustments [10-12]. Some of these devices have limited degrees of freedom preventing adequate adaptation for different test subjects sizes [2,13], while others are on a sloped physiological axis [2]. Some devices have a setting design which impedes the contraction [14]. These devices also have restrictions in their pre-stretching mechanism. Some have no pre-stretching mechanism at all, while others define the pre-stretching optimum by the stretching force not by the stretching length [10,12]. Devices which do assess the muscle length use graded mechanisms, either roughly graded [14] or with finer gradations obtained by using a step-motor [11,13], and have no variable pre-stretching mechanism. Additionally, the imprecise axle-adjustment and the constant extremity-setting lead to an ill-defined and variable contraction force vector in pre-stretching [11-14].

An ideal measurement device should be able to precisely determine and quantify a neuromuscular deficit. For clinical in-vivo measurements, a device as reproducible and valid as the in-vitro model is required. The device should be able to examine single muscles and to precisely define the physiological rotation axis of the limb, the force vector and the muscular length of an individual test subject. Furthermore, the optimum length of the muscle should be easy to determine and reproduce. All these conditions must be kept constant during the examination. To monitor progress of a disease and its response to treatment, it is also important to make sure that the sensitivity of the tests is sufficient to detect changes in the muscle's contraction. This implies that the variability in the measurements is known and it is small enough to allow changes to be recognized [15]. We suggest that our myograph [16] has all the characteristics described above, including those which are necessary to warrant a high standard for these clinical measurements.

The first aim of our study was to evaluate the quality of our newly developed myograph by assessing the reproducibility of the measurements of muscle force within and between testing sessions. The second aim was to determine reference values and the reference interval for the maximal force of the *adductor pollicis *indirectly stimulated with a single electrical supra-maximal pulse.

## Methods

### Device

The contraction force of musculus adductor pollicis was recorded using a specially developed myograph. This device was described in detail earlier [16]. Briefly, the core parts of the apparatus are the elements for axle-adjustment, for extremity-setting and for pre-stretching the muscle to its optimal length as well as the elements for stimulating the nerve, and for measuring muscle length and muscle force.

An extremity-setting module was chosen for the examination of the right *musculus adductor pollicis*. A laser beam running directly through the axle of the apparatus was projected onto the anatomical landmarks of the hand of the test subject, ensuring that physiological rotation axis was made congruent with the axle of the apparatus. When the hand was in the correct position, mounted cushion pads were moved towards arm and hand by electro-motors. A smoothly adjustable pre-stretching mechanism enabled the muscle to be pre-stretched to any physiological length, and then locked into place isometrically. Each position of the cushion pads and of the stopping mechanism was registered with electrical measurement recorders. This information was stored and the mechanic elements could then be moved automatically into position when that same test subject was examined again at a later time.

### Subjects, Place, Time

The study were carried out in accordance with the Helsinki Declaration of the World Medical Association and approved by the local Ethics Committee. All experiments were performed in the Department of Anesthesiology of Hannover Medical School. The first part of the study was carried out on 5 healthy voluntary test subjects, two women and three men. They were between 20 and 30 years old, the range of body height was 1.57 m to 1.96 m, the range of weight 45 kg to 103 kg. All of the test subjects were right handed, had no history of neuromuscular disorder or injury, and gave free, written informed consent to participate in this study. The second part of the study was performed on 405 healthy test subjects (aged 22–41). The contraction force was determined twice on each test subject.

### Protocol

Reproducibility: We examined 5 test subjects. For every subject, 100 datasets were collected. The examinations for each subject were performed over 10 sessions by three randomly selected investigators. There was an interval of 1 to 5 days between two consecutive sessions. The examinations started between 4 and 5 p.m. During each session an experiment was performed 10 times per test subject. To ensure the quality of the setting module of the device and to guarantee identical experimental conditions within and between the sessions, the extremity of the test subject was taken off the myograph after each recording. For each new recording, the extremity was replaced, readjusted and attached to the myograph. Furthermore, prior to each recording, the stimulation electrodes were placed again above the *nervus ulnaris*, the amplitude of the supra-maximal stimulus was determined and the optimal muscle length was noted down. Each experiment lasted approximately 20 minutes and the whole session 3 hours 20 minutes.

Reference interval: We examined 405 healthy test subjects. The experimental conditions were the same with the ones described for the reproducibility tests, except for a reduction in the number of force testings to two for each subject. Between the two examinations, the subject's extremity was reattached to the apparatus and the optimal muscle length was determined again.

### Stimulation and recordings

The *nervus ulnaris *was stimulated at the level of the wrist, using surface electrodes. For the study the *musculus adductor pollicis *was stimulated via the *ulnar nerve *at the level of the wrist. Using this stimulation site at the radial side, only two muscles are activated: the *musculus adductor pollicis *and *the flexor pollicis brevis*. Both muscles can be seen as a functional association with the same origin, same insertion and same physiological axis for the thumb movement. Their contractions are completely transmitted to the force sensor. At the ulnar side the *ulnar nerve *activates some other muscles too, their contractions, however, are buffered by the setting cushions and their contractions do not contribute to the force output which is measured. The stimulation was supra-maximal, with monophasic rectangular waveform, had a duration of 0.1 ms and an amplitude between 7 and 28 mA, thus ensuring that all motor units of the *musculus adductor pollicis *were simultaneously stimulated, once for each pulse. The recordings were carried out in the same laboratory and in the same setting, at a room temperature of 20–21°C.

### Data Analysis

Statistical analysis was performed using analysis of variance for both measurements within a testing session and between testing sessions. Post hoc discriminations were made with the Bonferroni significance correction using SPSS software. The reproducibility of muscle force was determined by two-way analysis of variance, which made it possible to discriminate between the force of the muscle tested, usual fluctuations during a test period and a real error of measurements.

Furthermore, a hierarchical variance analysis was carried out using the following linear model: y_ijk _= μ + p_i _+ d_ij _+ e_ijk_,

where: y_ijk _is the reproducibility of measured muscle force of observation ijk, μ is the overall mean, p_i _is the effect of test-person j, d_ij _is the effect of day ij (nested in test-person i) and e_ijk _is the residual effect connected with observation ijk.

Analyses were performed for consecutive numbers of observations from 10 to 2 within subclasses (test person, day). The GLM procedure of SAS was used.

## Results

**Figure 1:**
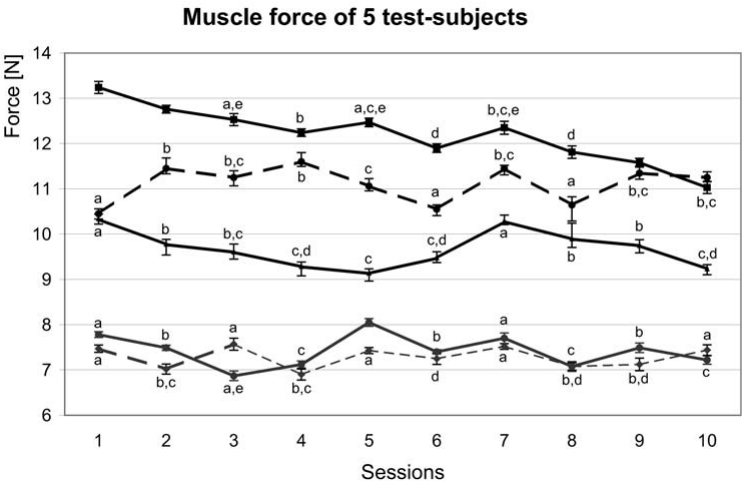
Muscle force (m. adductor pollicis) measured for 5 test subjects during 10 sessions. Every point represents the mean value of 10 measurements (± standard deviation). The mean muscle force obtained for test subjects varied between 12.2 N and 7.2 N. The results of the three upper curves belong to three male test subjects and are less consistent (12.2 – 11.1 N), in comparison with the muscle force registered for two female test subjects (mean values 6.87 and 8.05 N respectively). a, b, ... mean values marked by the same superscripts are not significantly different for the same test subject (P ≤ 0.05).

In the first part of the study, the muscle force calculated as a mean of 10 datasets collected during one session was 9.13 – 13.24 N for men and and 6.87 – 8.05 N for women (Fig. 1). The difference between the mean muscle force of tested men and women (3 and 2 subjects, respectively) was significant (P < 0.05). Furthermore, the results obtained from the female subjects were more homogenous during the test period, compared with the three tested men.

Changes of measured muscle force between consecutive sessions on each test subject were characterized using mean value and standard deviation and compared using analysis of variance. Interestingly, in some cases, in the 7^th ^session for men or in 5^th ^and 7^th ^sessions for women, an increase in the mean force of muscle contraction was found. In contrast to the increased variability of the mean muscle force between test-subjects, the muscle force for a given subject was very stable over the 10 sessions (Fig. 1). The maximal absolute differences were between 0.3 to 0.9 N in the men and 0.2 N in the two women.

**Figure 2:**
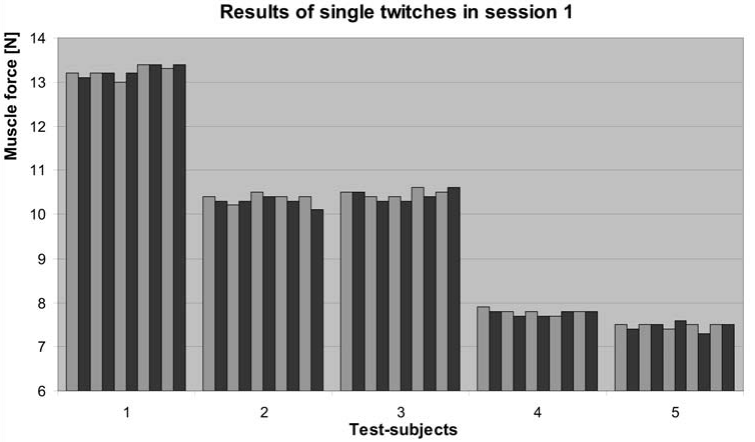
Reproducibility and variability of measured muscle force in five test subjects examined 10 times at short time intervals during the first session.

Important to notice is that the differences of muscle force for the same test subject measured during one session were small and not significant (Fig. 2). Thus the reproducibility of muscle force measured with the use of the myograph was accurate. To determine the precision of the device, standard deviations of results for each test-subject and for each session of 10 tests were determined and the mean value of all 50 standard deviations was calculated (0.12 N) and divided by the mean value of the force for all tests (9.53 N). The result, the coefficient of variation (0.0126), indicates the precision of the measurements (98.74%). Therefore, the whole error of the measurements performed with the newly developed myograph reaches only 1.3%.

Using a hierarchical variance analysis, the results obtained for every test subject during one session were homogenous enough to suggest that not more than two consecutive measurements are needed in order to assess a real, representative individual value of muscle force.

For the calculation of the reference interval, 810 force values from two consecutive measurements of 405 test subjects were obtained. The mean value was 9.51 N, the standard deviation +/- 2.42 N and the 95% reference interval was 4.77 – 14.25 N.

## Discussion

Precise quantitative measurements are necessary to assess neuromuscular deficit, while accurate and sensitive measurements are needed to document therapeutic efficacy and/or to define the natural history of a disease [4]. Additionally, to monitor the progress of a disease and its response to treatment, it is important to make sure that the sensitivity of the tests is high enough to allow changes to be detected. Clinicians normally rely on EMG or on manual testing to examine muscle functions. While the first method does not analyse muscle mechanics, the second method relies on subjective assessment by the clinician and is therefore inadequate to quantify small changes in muscle strength. Up to now, no known device for mechanical muscle testing, no matter if it uses voluntary or electrically stimulated contraction, can repeatedly examine the muscle at its individual optimal length, due to technical limitations of the setting and the pre-stretching mechanism [3-5,7].

Our myograph, however, can do this and may therefore examine an *in-vivo *muscle under the same precise conditions as a separated muscle *in-vitro *[16,17]. Its features include a reproducible extremity setting with precise axle adjustment, an infinitely adjustable pre-stretching mechanism, and the ability to maintain constant conditions during the experiment with a full transfer of the muscle force to the sensor.

The primary purpose of the study was to investigate the reproducibility of the results of measurements performed on our myograph. To this purpose, the muscle force of 5 young test subjects was assessed over 10 sessions of 10 tests each. The measurements were performed on the *musculus adductor pollicis*, which can be stretched in-vivo to its optimal length and is one of the key-muscles for clinical testing.

Focusing on the practical use of our myograph, we analyzed the variability of the muscle force measured within one session and between consecutive sessions on the same test subject. The reproducibility was so high, that only two consecutive measurements would be needed in order to collect objective data. The variability of muscle force, measured as standard deviation (SD), was on average lower than 0.12 N during 10 sessions, which results into a percentage change (expressed as SD × 100/mean) of only of 1.3% when compared with a mean muscle force of 9.53 N. Although objective comparison to devices described in the literature and those used in the clinical setting represents a difficult challenge, the results obtained with our myograph were more homogenous to those obtained with other devices [2,6,7].

The assessment of muscle force is often used to determine disease progression and, consequently, to assess the results of the therapeutic interventions. A high degree of reproducibility is essential for measurements across testing sessions to ease the monitoring of the disease progression. In our study, measurements of muscle force were very reproducible, confirming the quality and reproducibility of the results obtained with our myograph. The comparison to results described in literature is difficult, since different methods were used and their technical application allows fewer degrees of adjustment. Nevertheless, the precision of 98.64% in our measurements is higher than the precision described so far in literature for other devices. Brass et al. [11] described torque assessment of two muscle groups in humans. The variability of muscle force of *adductor pollicis*, calculated as the percentage difference between the second test relative to the first one was 4.6% ± 4.7% (n = 7). Wang et al. reported a variability of 7% during measurements of maximal force for the knee flexor and extensor within a single test session[18]. The authors indicated that this was due to difficulties in the standardization of the testing position and thus of the muscle length. Measurements of maximal voluntary torque by Todd et al. [2] also displayed a variability within a testing session (coefficient of variation range, 5–11%). In some tests described in the literature, the quality of measurements relied on the experience of the examiners and was more stable when subjects maintained some degree of physical activity [6]. This type of error for any measuring device has two components: inaccuracies of the measuring device and natural fluctuations in the force of contraction during the same session. Natural fluctuations occur for instance because surrounding muscular and non muscular structures lead to inhomogeneous sarcomer lengths, therefore the respective optimum muscle lengths and the respective number of actin-myosin connections in the muscle differ slightly between two consecutive examinations [19,20].

The substantial difference between our myograph and other devices is the possibility to precisely examine the individual muscle at its optimal length and to keep these conditions constant during the test. Thus, data obtained with the use of our myograph are valid independently of the special examination procedure and device.

In comparison to the other devices, the high precision of the newly developed myograph allows more efficient measurement procedures, so that studies can be carried out with fewer patients with less expense and also over a shorter time period. Only two consecutive measurements are required to obtain the same confidence as ten or more measurements performed during a session.

The precise, reproducible and valid measurements performed with our myograph and the successful definition of reference values opens up a clinical access to the etiology and the pathogenesis of nerve-muscle diseases. The progression of the disease may be more closely monitored and our device is sensitive enough to detect small differences or early deficits. If a general trend is defined, estimations of the prognosis may be obtained. There is even the possibility that therapies could be modified based on the test results. Clinical studies with a prototype of this myograph have begun on our intensive care unit. We monitor partly concious, partly unconcious patients with either a critical illness polyneuropathy or myopathy. Because this disease affects many skeletal muscles we can take the *musculus adductor pollicis *as a key muscle for the disease[21,22]. In diseases that affect only a few muscles, we have to choose an appropriate setting module for the selected muscle [16].

## Conclusion

The new myograph is a highly reliable measuring device with which the *adductor pollicis *can be investigated at the optimum length. The results of this study on healthy subjects provide a basis for the examination of nerve and muscle function on patients. A study protocol can be restricted to two consecutive measurements to assess the function at a specific time point. The assessed physiological reference interval may help to rate the grade of disability.

## Competing interests

NRM developed the device and is owner of two international patents. The authors declare that they have no other competing interests.

## Authors' contributions

NRM developed the device. NRM, MW, CW, and MG carried out the experiments, MW and SP co-ordinated the testing. MP helped with the physiological interpretation of the data. SP and AIA helped to draft the manuscript, NRM and MP wrote the manuscript.

## References

[B1] de Carvalho M, Lopes A, Scotto M, Swash M (2001). Reproducibility of neurophysiological and myometric measurement in the ulnar nerve-abductor digiti minimi system. Muscle Nerve.

[B2] Todd G, Gorman RB, Gandevia SC (2004). Measurement and reproducibility of strength and voluntary activation of lower-limb muscles. Muscle Nerve.

[B3] Colombo R, Mazzini L, Mora G, Parenzan R, Creola G, Pirali I, Minuto G (2000). Measurement of isometric muscle strength: a reproducibility study of maximal voluntary contraction in normal subjects and amyotrophic lateral sclerosis patients. Med Eng Phys.

[B4] Andres PL, Skerry LM, Thornell B, Portney LG, Finison LJ, Munsat TL (1996). A comparison of three measures of disease progression in ALS. J Neurol Sci.

[B5] Beck M, Gies R, Würffel W, Magnus T, Ochs G, Toyka KV (1999). Comparison of maximal voluntary isometric contraction and drachman's hand-held dynamometry in evaluating patient with amyotrophic lateral sclerosis. Muscle Nerve.

[B6] Morris-Chatta R, Buchner DM, de Lateur BJ, Cress E, Wagner EH (1994). Isokinetic testing of ankle strength in older adults: assessment of inter-rater reliability and stability of strength over six month. Arch Phys Med Rehabil.

[B7] Goonetilleke A, Modarres-Sadeghi H, Guiloff RJ (1994). Accuracy, reproducibility, and variability of hand-held dynamometry in motor neuron disease. J Neurol Neurosurg Psychiatry.

[B8] Sansone V, Marinou K, Salvucci J, Meola G (2000). Quantitative myotonia assessment: an experimental protocol. Neurol Sci.

[B9] Akataki K, Mita K, Itoh Y (1999). Repeatability study of mechanomyography in submaximal isometric contractions using coefficient of variation and intraclass correlation coefficient. Electromyogr Clin Neurophysiol.

[B10] Bellemare F, Couture J, Donati F, Plaud B (2000). Temporal relation between acoustic and force responses at the adductor pollicis during non-depolarizing neuromuscular block. Anesthesiology.

[B11] Brass TJ, Loushin MKH, Day JW, Iaizzo PA (1996). An improved method for muscle force assessment in neuromuscular disease. J Med Eng Technol.

[B12] McLaughlin SJ, Chan ST, Ford D, Neame SJ, Dudley HA (1987). Microcomputer system for the analysis of muscle function. J Biomed Eng.

[B13] Lee HD, Herzog W (2003). Force depression following muscle shortening of voluntarily activated and electrically stimulated human adductor pollicis. J Physiol.

[B14] Rutherford OM, Jones DA (1988). Contractile properties and fatiguability of the human adductor pollicis and first dorsal interosseus: a comparision of the effects of two chronic stimulation patterns. J Neurol Sci.

[B15] Olney RK (1998). Neurophysiological evaluation and clinical trials for neuromuscular diseases. Muscle Nerve.

[B16] Rahe-Meyer N, Weilbach Ch, Karst M, Pawlak M, Aminul A, Piepenbrock S, Winterhalter M (2007). In vivo myograph measurement of muscle contraction at optimal length. BioMed Eng OnLine.

[B17] Rahe-Meyer N, Winterhalter M, Gross M, Zuk J, Piepenbrock S (2005). Complex Myograph: evaluation of a diagnostic device and standard values of force, velocity and working capacity of the muscle in-vivo. Abstract. Europ J Anaesth.

[B18] Wang C-Y, Olson SL, Protas EJ (2002). Test-retest strength reliablility: hand-held dynamometry in community-dwelling elderly fallers. Arch Phys Med Rehabil.

[B19] Yucesoy CA, Koopman BH, Baan GC, Grootenboer HJ, Huijing PA (2003). Effects of inter- and extramuscular myofascial force transmission on adjacent synergistic muscles: assessment by experiments and finite-element modeling. J Biomech.

[B20] Huijing PA, Baan GC (2003). Myofascial force transmission: muscle relative position and length determine agonist and synergist muscle force. J Appl Physiol.

[B21] Leuwer M, Meyer N, Zuzan O, Nowak R (1999). Possible causes of weaning failure in critical illness. A novel aspect [abstract]. Brit J Anaesth.

[B22] Leuwer M, Kubis HP, Zuzan O, Meyer N, Nowak R, Scheumann G, Rueckoldt H (1999). Weaning failure in two critically ill patients is associated with changes in myosin subtypes [abstract]. Brit J Anaesth.

